# Inter-Population Variability of Endosymbiont Densities in the Asian Citrus Psyllid (*Diaphorina citri* Kuwayama)

**DOI:** 10.1007/s00248-016-0733-9

**Published:** 2016-02-04

**Authors:** Chia-Ching Chu, Torrence A. Gill, Mark Hoffmann, Kirsten S. Pelz-Stelinski

**Affiliations:** Citrus Research and Education Center, University of Florida, Lake Alfred, FL USA; Department of Entomology and Nematology, University of Florida, Gainesville, FL USA; Department of Plant Sciences, University of California, Davis, Salinas, CA USA

**Keywords:** Huanglongbing, Intracellular endosymbionts, Primary endosymbionts, Bacteriome

## Abstract

The Asian citrus psyllid (*Diaphorina citri* Kuwayama) is an insect pest capable of transmitting *Candidatus* Liberibacter asiaticus (*C*Las), the causal agent of citrus greening in North America. *D. citri* also harbors three endosymbionts, *Wolbachia*, *Candidatus* Carsonella ruddii, and *Candidatus* Profftella armatura, which may influence *D. citri* physiology and fitness. Although genomic researches on these bacteria have been conducted, much remains unclear regarding their ecology and inter-population variability in *D. citri*. The present work examined the densities of each endosymbiont in adult *D. citri* sampled from different populations using quantitative PCR. Under field conditions, the densities of all three endosymbionts positively correlated with each other, and they are associated with *D. citri* gender and locality. In addition, the infection density of *C*Las also varied across populations. Although an analysis pooling *D. citri* from different populations showed that *C*Las-infected individuals tended to have lower endosymbiont densities compared to uninfected individuals, the difference was not significant when the population was included as a factor in the analysis, suggesting that other population-specific factors may have stronger effects on endosymbiont densities. To determine whether there is a genetic basis to the density differences, endosymbiont densities between aged *C*Las-negative females of two *D. citri* populations reared under standardized laboratory conditions were compared. Results suggested that inter-population variability in *Wolbachia* infection density is associated with the genotypes of the endosymbiont or the host. Findings from this work could facilitate understanding of *D. citri*-bacterial associations that may benefit the development of approaches for managing citrus greening, such as prevention of *C*Las transmission.

**Electronic supplementary material:**

The online version of this article (doi:10.1007/s00248-016-0733-9) contains supplementary material, which is available to authorized users.

## Introduction

Bacterial endosymbionts of insects can profoundly influence their host’s biology [1–3]. Some bacteria provide nutritional or protective benefits to the host, while others can affect an insect vector’s efficiency in transmitting viruses [4–8]. Such findings suggest that insect-microbe associations may be exploited for managing insect pests or insect-transmitted diseases.

Citrus greening is a destructive plant disease caused by the Alphaproteobacterium *Candidatus* Liberibacter asiaticus (*C*Las) and other closely related species [9–12]. The Asian citrus psyllid (*Diaphorina citri* Kuwayama) transmits *C*Las in North America, Asia, and Brazil [10]. The sap-sucking *D. citri* can acquire *C*Las while feeding on infected citrus. After entering the vector insect, the bacteria can colonize and propagate in its digestive tract. Thereafter, the pathogen may cross the gut barrier and invade the salivary gland, thereby enabling *D. citri* to transmit the pathogen to another plant [13, 14]. In addition to this host-microbe association, *D. citri* also harbors three endosymbionts: (1) a species of *Wolbachia*, a group of Alphaproteobacteria commonly found in arthropod species [2, 7, 8, 15, 16], (2) the Gammaproteobacterium *Candidatas* Carsonella ruddii, an endosymbiont which may provide nutritional benefits to its host [17, 18] and (3) *Candidatus* Profftella armatura, a Betaproteobacterium capable of producing a defensive polyketide (diaphorin) [19]. In addition to their potential influence on *D. citri* biology, Profftella and *Wolbachia*’s relative abundances in *D. citri* correlate with abundance of *C*Las [20], suggesting that uncharacterized, direct or indirect, interactions may exist among *C*Las and these endosymbionts.

Infection density contributes to an endosymbiont’s influence on its host [21]. Previous studies on *D. citri* endosymbionts have either measured the endosymbionts’ relative abundance (rather than absolute densities), compared endosymbiont abundance without normalization against *D. citri* DNA, focused on single populations or only targeted one endosymbiont species [16, 20, 22]. Thus, much of the associations among endosymbionts, their host, and other ecological factors remain unclear. Characterizing host-microbe associations in field *D. citri* is important not only for understanding its general biology but also for application purposes; the possibility of exploring host-bacterial interactions for *C*Las control (i.e., paratransgenic approaches) has been proposed and studied [23, 24]. Interestingly, a previous study showed that *Wolbachia* density could vary among field populations of *D. citri* [16]. Although we could not rule out the possibility that *D. citri* age and gender contribute to such variation [22], it is unknown whether other population-specific factors (i.e., *C*Las infection, genetic variation) are also associated with these patterns and whether other *D. citri* endosymbionts may exhibit similar density variability across populations.

The present study investigated whether insect-microbe associations vary across *D. citri* populations by comparing the densities of *Wolbachia*, Carsonella, and Profftella in *D. citri* adults sampled from three locations in Florida. This research also hypothesized that either: (1) high endosymbiont densities exclude *C*Las in *D. citri*, or (2) endosymbiont presence facilitates colonization of *D. citri* by *C*Las. To evaluate these hypotheses, the effects of gender, population (locality), and infection by *C*Las on endosymbionts densities was examined. Alternatively, this study also investigated (3) whether genetic differences among *D. citri* or endosymbiont populations contribute to the inter-population variability of endosymbiont densities. These variations were evaluated using aged *D. citri* cultures from geographically separated populations reared under standardized conditions. Results from this study indicated that the densities of all three endosymbionts are population dependent, and that there is a genetic basis to the population effect on *Wolbachia* density. *C*Las infection in *D. citri* was also found more associated with the population differences than with the endosymbiont densities. Findings from this study could improve general and applied understanding of *D. citri* biology to manage the worldwide and devastating citrus greening disease.

## Methods

### Insect Materials

To investigate endosymbiont densities in field *D. citri*, adult insects (*n* = 123) were sampled from three locations in Florida: LaBelle (26°41′37.0″N, 81°27′22.3″W), Lake Alfred (28°05′42.0″N, 81°43′19.2″W), and Clermont (28°39′41.8″N, 81°44′48.4″W) during June 2015 (Table 1). To prevent possible influence of extreme temperatures on *D. citri* endosymbiont growth, we avoided exposing the insects to sunlight or keeping them on ice during transportation to the laboratory. After determining each *D. citri* gender, individuals were separately placed in microcentrifuge vials, flash frozen with liquid nitrogen, and stored at −80 °C.

**Table 1:** Details of Florida *Diaphorina citri* populations used in this study

Population	Sampling site	Total *D. citri* sampled	Females	Males
LaBelle (field)	26°41′37.0″N, 81°27′22.3″W	42	18	24
Lake Alfred (field)	28°05′42.0″N, 81°43′19.2″W	41	14	27
Clermont (field)	28°39′41.8″N, 81°44′48.4″W	40	17	23
Lake Alfred (colony)	Laboratory	12	12	0
Clermont (colony)	Laboratory	10	10	0

Comparisons of endosymbiont densities were made between two laboratory cultures collected from Lake Alfred and Clermont Florida (Table 1) in order to control for variation associated with age, diet, and environment. The two cultures were reared on *Murraya koenigii* for over 2 months (approximately three generations) to reduce the proportion of *C*Las-infected psyllids. *M. koenigii* is a host plant of *D. citri*, but not *C*Las [25]. Adult *D. citri* from these colonies were allowed to oviposit on ‘Swingle’ citrumelo [*Citrus paradisi* MacFaden × *Poncirus trifoliata* (L.) Raf.] placed in separate screen cages (24 × 12 × 12 in.) in a greenhouse at 28 ± 1 °C, 50 ± 10 % RH to reflect typical field conditions. The original (parent) *D. citri* adults were removed following egg hatch. One-day-old adult female offspring were sampled daily over for 6 days. Females were sampled because they tend to exhibit greater inter-population difference in endosymbiont densities compared to males, as described below. Each individual was flash frozen and stored at −80 °C as described above. Laboratory-reared insects that were not infected with *C*Las (Lake Alfred, *n* = 12; Clermont, *n* = 10; Table 1) were used in the experiments described below.

DNA from each sample was extracted following a brief wash with 70 % alcohol using the DNeasy Blood & Tissue Kit (QIAGEN, Inc., Valencia, CA). DNA samples were quantified using a NanoDrop 2000 spectrophotometer (Thermo Fisher Scientific, Lafayette, CO) and diluted to 15 ng μl^−1^ for subsequent quantitative real-time polymerase chain reaction (qPCR) analysis.

### Construction of qPCR Standard Curves

Template DNA was prepared for conserved genes of *D. citri* and each of its endosymbionts, including the *D. citri wingless* gene, the 16S rDNA of *C*Las, Profftella, and Carsonella, and the *Wolbachia ftsZ* gene (Table 2). To obtain DNA templates required for qPCR standard curves, plasmids containing the targeted gene fragments were constructed. 16S rDNA and *ftsZ* gene fragments were amplified using primers and PCR conditions described previously (Table 2) [22, 26–28]. Gene fragments were cloned into the pGEM-T easy vector (Promega, Inc., Madison, WI, USA) and transformed into *E. coli* strain JM109 following the manufacturer’s instructions. Plasmids for the *C*Las 16S rDNA and *D. citri wingless* gene were prepared as described in Coy et al. [26] using pGEM-T easy vectors. Plasmids were purified from overnight cultures of transformed JM109 strains using the QIAprep Spin Miniprep Kit (QIAGEN, Inc., Valencia, CA). Afterwards, 1 μg of each plasmid was digested using PstI (New England Biolabs, Inc., Beverly, MA) following the manufacturer’s instructions (in 50 μl reactions). Linearized plasmids were purified from the digestion reactions using the QIAquick PCR Purification Kit (QIAGEN, Inc., Valencia, CA) and underwent serial dilutions for qPCR standard curve assays.

**Table 2:** Details of primers or probes used for qPCR assays included in this study

Target species	Target gene	Assay type	Primer/Probe sequence	Reference
*Candidatus* Liberibacter asiaticus	16S rDNA	Taqman	TCGAGCGCGTATGCGAATAC	[26, 27]
			GCGTTATCCCGTAGAAAAAGGTAG	
			AGACGGGTGAGTAACGCG	
*Diaphorina citri*	*wingless*	Taqman	GCTCTCAAAGATCGGTTTGACGG	[28]
			GCTGCCACGAACGTTACCTTC	
			TTACTGACCATCACTCTGGACGC	
*Candidatus* Carsonella ruddii	16S rDNA	SYBR	TGGGAACGCCATATGCTAAT	[22]
			GTCCCAATGGGTTGTTCATC	
*Candidatus* Profftella armatura	16S rDNA	SYBR	GCCTTTATGGGTAGGGCTTC	[22]
			CCGGACTACGATGCACTTTT	
*Wolbachia*	*ftsZ*	SYBR	AGCAGCCAGAGAAGCAAGAG	[22]
			TACGTCGCACACCTTCAAAA	
*Wolbachia*	*wsp*	Nested PCR	TGGTACAATAAGTGATGAAGAAAC	[15]
		(outer)	AAAAATTAAACGCTACTCCA	
		Nested PCR	GGATAGTCCCTTAACAAGAT	
		(inner)	TTGATTTCTGGAGTTACATC	

### Quantitative PCR Assays

Both the Taqman probe (*C*Las and *wingless*, multiplex qPCR) and SYBR green methods (Carsonella, Profftella, *Wolbachia*) were used in this work (Table 2). To assure that quantifications of the same target genes were comparable across different qPCR plates, all samples were analyzed using standard curves constructed from the same preparation of linearized plasmid template. Quantitative PCR assays were conducted using an Applied Biosystems 7500 Fast Real-Time PCR System and the SYBR Green PCR Master Mix (Applied Biosystems, Inc., Foster City, CA) or the PerfeCTa qPCR ToughMix, Low ROX (Quanta BioSciences, Inc., Gaithersburg, MD; for Taqman assays). Final primer and probe concentrations were 0.2 μM (for Carsonella and Profftella measurements) or 0.1 μM (for *wingless*, *C*Las and *Wolbachia* assays). One microliter (15 ng) of *D. citri* template DNA was used in a 25 μl qPCR reaction volume. Three technical replicates were performed for each sample and standard curve DNA reaction. The qPCR conditions consisted of one cycle of 95 °C for 10 min, 40 cycles of 95 °C for 15 s and 58 °C (Carsonella and *Wolbachia*) or 60 °C (Profftella, *C*Las and *wingless*) for 30 s. Dissociation curve analyses were conducted for SYBR green assays. PCR efficiencies were confirmed to be within the range of 90–110 % (*R*^2^ > 99 %) for all qPCR assays. The melting curves for all of the SYBR green assays indicated that only the correct amplicons were amplified and no detectable primer dimers were produced.

### Data Analysis

For each sample, the copy numbers of the five target genes in the template DNA were calculated using a previously described method [29]. Subsequently, the endosymbiont copy number was divided by the *wingless* gene copy number in the same sample. Many of the *D. citri* samples had extremely low *C*Las infection levels and were therefore difficult to accurately quantify using standard curves. Thus, *C*Las density across samples was compared by dividing infected *D. citri* into *high infection* and *low infection* samples using a density threshold (0.02 copies of *C*Las 16S rDNA per copy of *wingless*). Correlations among the densities of different endosymbionts and the effects of locality, gender, and *C*Las infection on endosymbiont densities were determined/analyzed using SPSS 23 (IBM, Inc.). Statistical analyses (correlation analysis, chi-square test of homogeneity, analysis of variance) were conducted on the endosymbiont densities (measured as *copies of endosymbiont per copy of wingless*). Endosymbiont densities among field-collected *D. citri* were transformed for statistical analysis using *log(1 + x)* (*Wolbachia*) or *log* (Carsonella and Profftella) to satisfy the condition of normality for analysis of variance (ANOVA). In some ANOVA tests where the test assumptions were violated after data transformation, a more stringent significant threshold was used (*P* < 0.01); significant factors in these tests were only reported if they meet this threshold. In ANOVA tests where test assumptions were satisfied or only mildly violated, the significant factors were reported when *P* < 0.05.

## Results

### Presence and Densities of Different Endosymbionts in Field *D. citri* Samples

**Fig. 1:**
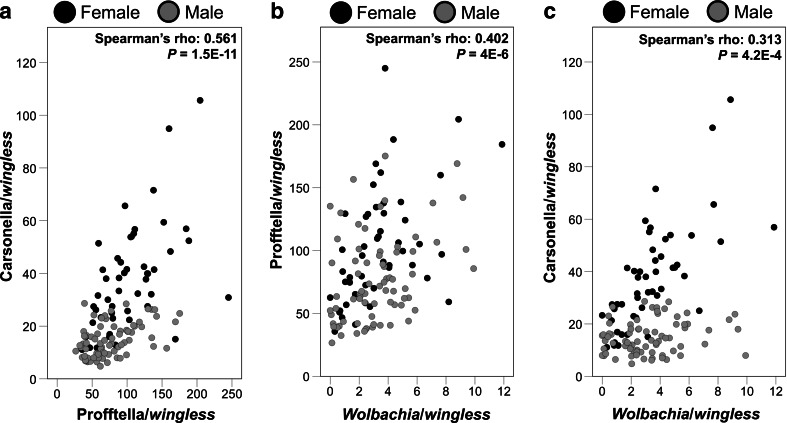
Scatter plots illustrating correlations among densities of *D. citri* endosymbionts. **a** Carsonella and Profftella, **b** Profftella and *Wolbachia*, **c** Carsonella and *Wolbachia*. Observations are colored (*black* or *gray*) by *D. citri* gender. Correlations of the endosymbiont densities in each plot were determined using pooled data from both genders. Correlation coefficients of endosymbiont densities in female *D. citri* were 0.594 (*P* = 7.0E−6) for Carsonella and Profftella, 0.561 (*P* = 2.8E−5) for Profftella and *Wolbachia*, and 0.75 (*P* = 5.5E−10) for Carsonella and *Wolbachia*. Correlation coefficients in males were 0.427 (*P* = 1.5E−4) for Carsonella and Profftella, 0.334 (*P* = 0.004) for Profftella and *Wolbachia*, and 0.2 (*P* = 0.088) for Carsonella and *Wolbachia*

**Fig. 2:**
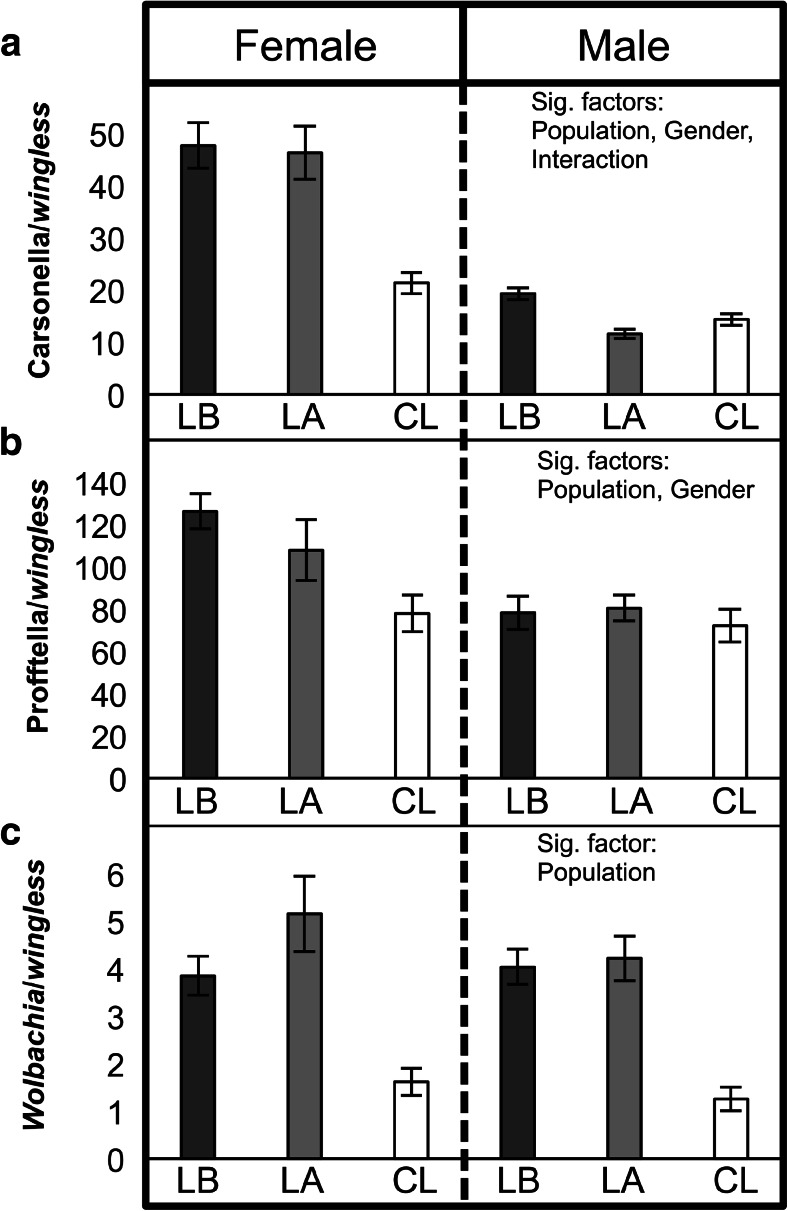
Densities of **a** Carsonella, **b** Profftella, and **c** *Wolbachia* in *D. citri* adults from different field populations. *LB* LaBelle; *LA* Lake Alfred; *CL* Clermont. Factors significantly associated with each endosymbiont’s density are shown on the *right*. The *error bars* indicate standard errors. *LB* LaBelle; *LA* Lake Alfred; *CL* Clermont

Carsonella and Profftella were detected among all 123 *D. citri* tested. Only three individuals tested negative for the presence of *Wolbachia*: one male and one female from Lake Alfred, and one male from Clermont. Additional testing of these three samples using a previously described nested conventional PCR assay [15] also failed to detect the endosymbiont (Fig. S1). Overall, Carsonella, Profftella, and *Wolbachia* were positively correlated with each other (*P* < 0.05; Fig. 1). Positive correlations among the endosymbionts were also detected within each *D. citri* gender, with the exception of Carsonella and *Wolbachia* in male insects (*P* = 0.088; Fig. 1c). The densities of all three endosymbionts were population dependent (Carsonella: *F* = 22.4, df = 2, 117, *P* < 0.0001; Profftella: *F* = 6.02, df = 2, 117, *P* = 0.0032; *Wolbachia*: *F* = 39.49, df = 2, 117, *P* < 0.0001). The Clermont population exhibited the lowest infection densities as compared with the Lake Alfred and LaBelle populations (Fig. 2). *D. citri* gender was significantly associated with the densities of Carsonella and Profftella (Carsonella: *F* = 163.02, df = 1, 117, *P* < 0.0001; Profftella: *F* = 15.61, df = 1, 117, *P* = 0.0001). Infection densities were higher in females as compared to male *D. citri* (Fig. 2). *Wolbachia* densities were not significantly associated with *D. citri* gender (*P* > 0.05).

### Infection Prevalence and Density of CLas Among Field *D. citri*

**Fig. 3:**
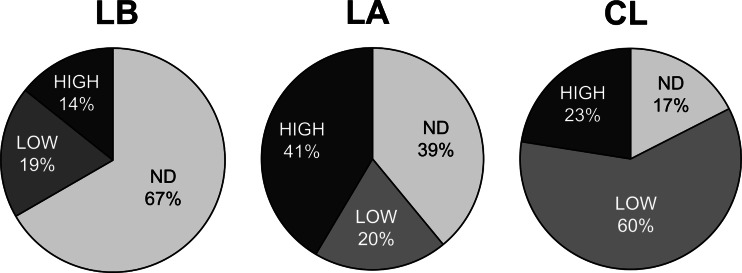
Proportions of *D. citri* infected with different densities of *C*Las. *ND* No *C*Las detected; *LOW C*Las densities lower than 0.02 copy of *C*Las 16S rDNA per copy of *wingless. HIGH C*Las density higher than the threshold. *LB* LaBelle; *LA* Lake Alfred; *CL* Clermont

Among the 123 field *D. citri* adults sampled, 72 individuals tested positive for the presence of *C*Las. The three *Wolbachia*-negative individuals (described above) all carried *C*Las. Cross-population comparisons of the *C*Las measurements revealed that both the prevalence and the density of *C*Las infection were population dependent (*X*^*2*^ = 31.97, df = 4, *P* < 0.05; Fig. 3). Individuals from the LaBelle population had the lowest *C*Las infection rate while those from the Clermont population had the highest (Fig. 3). Although the Lake Alfred population had fewer *C*Las-positive individuals than the Clermont population, there was a higher proportion of *D. citri* infected with high densities of *C*Las in the Lake Alfred population (high and low infection samples only, Lake Alfred vs Clermont, *X*^*2*^ = 9.54, df = 1, *P* < 0.05). When comparing *C*Las densities across population within each gender, similar patterns were observed (Fig. S2).

### Associations Between *C*Las Infection and Endosymbiont Densities

**Fig. 4:**
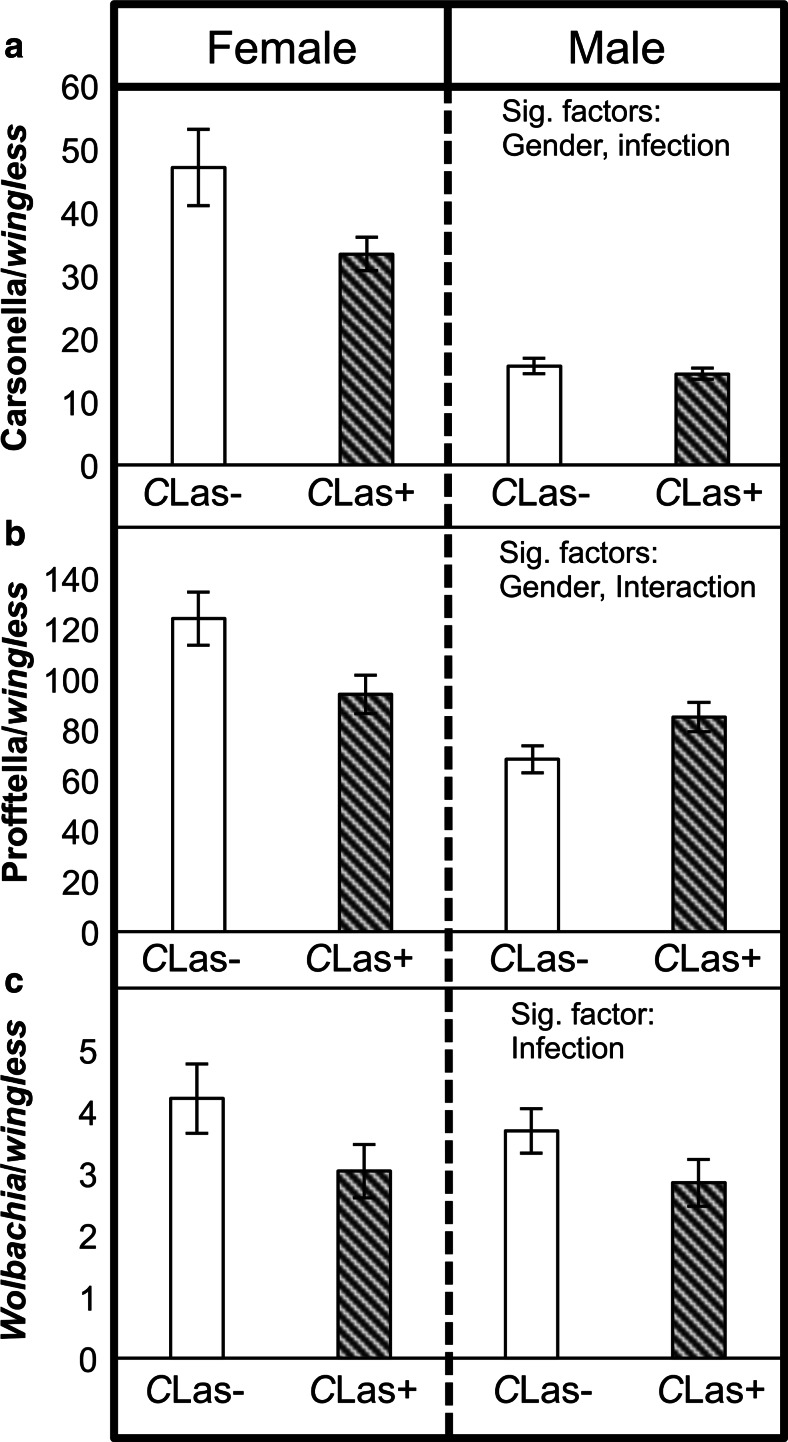
Densities of **a** Carsonella, **b** Profftella, and **c** *Wolbachia* in *C*Las-infected (*C*Las+) or uninfected (*C*Las−) field *D. citri* (total *n* = 123). Factors significantly associated with each endosymbiont’s density are shown on the right. The *error bars* indicate standard error

To investigate associations between *C*Las infection and the densities of *D. citri* endosymbionts, *D. citri* samples were categorized according to their *C*Las infection status (positive or negative) and their endosymbiont densities were compared (Fig. 4). The densities of Carsonella and *Wolbachia* were significantly higher in *C*Las-negative compared to *C*Las-positive *D. citri* when 123 samples were pooled for two-way ANOVA using gender and infection status as fixed factors (Carsonella: *F* = 4.96, df = 1, 119, *P* = 0.0278; *Wolbachia*: *F* = 7.97, df = 1, 119, *P* = 0.0056). There was also a significant interaction effect for *gender* and *infection status* on Profftella density (Fig. 4) (*F* = 10.5, df = 1, 119, *P* = 0.0015); *C*Las-infected females had a lower density of Profftella as compared to uninfected females, while the opposite pattern was observed in male *D. citri* (Fig. 4b). When analyzed in a three-way ANOVA (population × gender × infection status), there was a significant population effect across all three endosymbionts (Carsonella: *F* = 15.96, df = 2, 111, *P* < 0.0001; Profftella: *F* = 5.1, df = 2, 111, *P* = 0.0076; *Wolbachia*: *F* = 26.5, df = 2, 111, *P* < 0.0001), yet the effect of the *C*Las infection status was no longer significant (*P* > 0.05). When categorizing samples based on the *C*Las infection density (low, high, or ND) instead of their infection status, the *ND* (no *C*Las detected) females tended to have higher endosymbiont densities than the *low* and *high* infected females (Table S1). Similar to *infection status*, the effect of *C*Las *infection density* on endosymbiont densities was not significant (*P* > 0.05) when the data were analyzed using a three-way ANOVA including the *population* factor. Details of the endosymbiont density values can be found in the supplementary material (Table S1).

### Endosymbiont Densities in *D. citri* Populations Reared Under Standardized Conditions

**Fig. 5:**
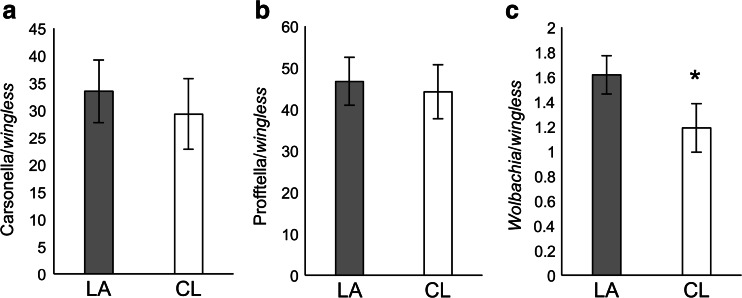
Densities of **a** Carsonella, **b** Profftella, and **c** *Wolbachia* in laboratory-reared *D. citri* populations. *LA* Lake Alfred population; *CL* Clermont population. The *asterisk* indicates significant difference between populations (one-tailed *t* test *P* < 0.05). The *error bars* indicate standard errors

Laboratory *D. citri* cultures exhibited *Wolbachia* densities similar to the patterns observed among field *D. citri* populations. *Wolbachia* densities were higher in the Lake Alfred-derived *D. citri* culture compared to the Clermont *D. citri* culture (one-tailed *t* test; *t* = 1.73, df = 20, *P* < 0.05; Fig. 5). The densities of Carsonella and Profftella did not differ significantly between the *D. citri* cultures from these locations (Fig. 5).

## Discussion

Characterizing factors associated with infection densities of *D. citri* endosymbionts is a necessary step for understanding these bacteria’s influence on the psyllid host. Using qPCR and absolute quantitation methods, the present study investigated the ecology of *D. citri* endosymbionts across different field populations. The densities of *Wolbachia*, Profftella, and Carsonella were positively correlated with each other among the field *D. citri* populations, indicating that the growth of an individual endosymbiont is not significantly inhibited by the growth of others. Among the three endosymbionts, there was a stronger correlation (higher coefficient, lower *P* value) between the densities of Profftella and Carsonella. It is possible that the growth of Profftella and Carsonella, which both inhabit the bacteriomes of *D. citri*, may be more synchronized with each other than with *Wolbachia*. Unexpectedly, even when assessed using the most sensitive nested-PCR assay, *Wolbachia* was not detectable in three out of the 123 *D. citri* analyzed. Previous studies reported 100 % *Wolbachia* infection among *D. citri* populations in Florida and Brazil [15, 16]; however, the present work suggests that rare failures of vertical transmission may occur in the field. Examining larger sample sizes or sampling insects during different times of the year may be necessary to detect their occurrence. The finding of *D. citri* individuals infected with *C*Las, but not *Wolbachia*, also suggests that *Wolbachia* infection is not a requirement for *C*Las acquisition.

The higher density of Profftella and Carsonella in *D. citri* females may reflect these bacteria’s higher rate of abundance increase with age in females relative to males [22]. It is therefore possible that such effects could contribute to the positive correlations and between-gender differences among endosymbiont densities observed in the field samples.

Although the *D. citri* gender and age could be factors affecting the endosymbionts’ densities, it does not completely explain the inter-population variability observed among the field *D. citri* samples. For example, if they were the sole factors determining endosymbiont density, one would expect that the density differences among populations (within gender) would be consistent across all three endosymbionts, since all three endosymbiont densities should gradually increase as their host age [22]. Instead, the data showed that while Carsonella and Profftella densities were similar among the males from all three populations tested, the *Wolbachia* densities were variable. The *C*Las infection status of *D. citri* and genotypic variations in *D. citri* and its endosymbionts are possible factors associated with endosymbiont density. Although the possibility that unique endosymbiont-*D. citri* associations (originating from genetic differences) across populations might influence *D. citri*’s efficiency to acquire *C*Las could not be ruled out, the present study falsified the hypotheses that high endosymbiont densities per se either exclude or facilitate *C*Las acquisition in *D. citri* (i.e., suggesting that there is no competitive exclusion occurring among endosymbionts and *C*Las). The results indicated that, although an analysis pooling *D. citri* from different populations suggested that *C*Las-infected individuals tend to have lower endosymbiont densities compared to uninfected individuals, the same difference was not observed when the population factor was considered (three-way ANOVA model). This finding suggests that population-specific factors, such as genetic background, may have a stronger effect on the endosymbiont densities.

Interestingly, we found that differences between *Wolbachia* densities of the Lake Alfred and Clermont *D. citri* were consistent between field samples and those reared under laboratory conditions (standardized diet, age, and environment), which supports the hypothesis of a genetic basis underlying *Wolbachia* density. In other insect systems, genotypes of the insect and bacteria can determine *Wolbachia* infection density [21, 30]. The present study indicates that host-endosymbiont interactions could vary among field *D. citri* populations and may have different repercussions on their biology.

*C*Las infection prevalence and density varied among the *D. citri* populations tested. A recent study indicated that *D. citri* from southern Florida (e.g., LaBelle) had lower *C*Las infection prevalence compared to northern Florida populations (e.g., Lake Alfred) [31]. Here, the present study not only detected similar differences in the *C*Las infection prevalence among the three Florida populations, but also showed that the infection intensity also differ among them. A systemic infection by *C*Las, followed by invasion of the salivary gland could be necessary for *D. citri* to become capable of transmitting the bacteria to healthy plants [14]. A recent study also suggested that *C*Las density in *D. citri* correlates to their transmission efficiency [32]. Therefore, determining both the density and prevalence of *C*Las infection in field *D. citri* may allow more accurate monitoring of citrus greening in the field.

Overall, findings from this work indicate that variations between *D. citri* populations appear more important than endosymbiont density in predicting *C*Las infection. Recognizing the heterogeneous host-microbe interactions in field *D. citri* and different *C*Las infection densities across populations could provide venues for investigating *D. citri* biology and developing strategies for citrus greening management, such as methods to intervene in the pathogen transmission process.

## Electronic supplementary material

Below is the link to the electronic supplementary material.

Figure S1 Nested PCR targeting a 354 bp fragment (arrow) of the *Wolbachia* surface protein gene (*wsp*) did not detect *Wolbachia* in three of the field-sampled ACP adults. L: 100 bp ladder; P: positive control (genomic DNA of a *Wolbachia*-positive ACP) amplified using the inner primer pair only; P-*n*: positive control, after two rounds of amplification; ACP1-3: *Wolbachia*-negative ACP samples (determined by qPCR assays), after two rounds of PCR; N: no template control, tested using inner primers only; N-*n*: no template control, after two rounds of PCR. (GIF 104 kb)

High resolution image (TIFF 5980 kb)

Figure S2 Proportions of female and male *D. citri* infected with different densities of *C*Las. ND: No *C*Las detected; LOW: *C*Las densities lower than 0.02 copy of *C*Las 16S rDNA per copy of *wingless*. HIGH: *C*Las density higher than the threshold. LB: LaBelle; LA: Lake Alfred; CL: Clermont. (GIF 1930 kb)

High resolution image (EPS 848 kb)

Table S1 (DOC 63 kb)
