# Supplementary figures and images for: Inter-Population Variability of Endosymbiont Densities in the Asian Citrus Psyllid (Diaphorina citri Kuwayama)
Source: Microb Ecol. 2016 Feb 4;71:999–1007. doi: 10.1007/s00248-016-0733-9 (PMC4944574; doi:10.1007/s00248-016-0733-9)

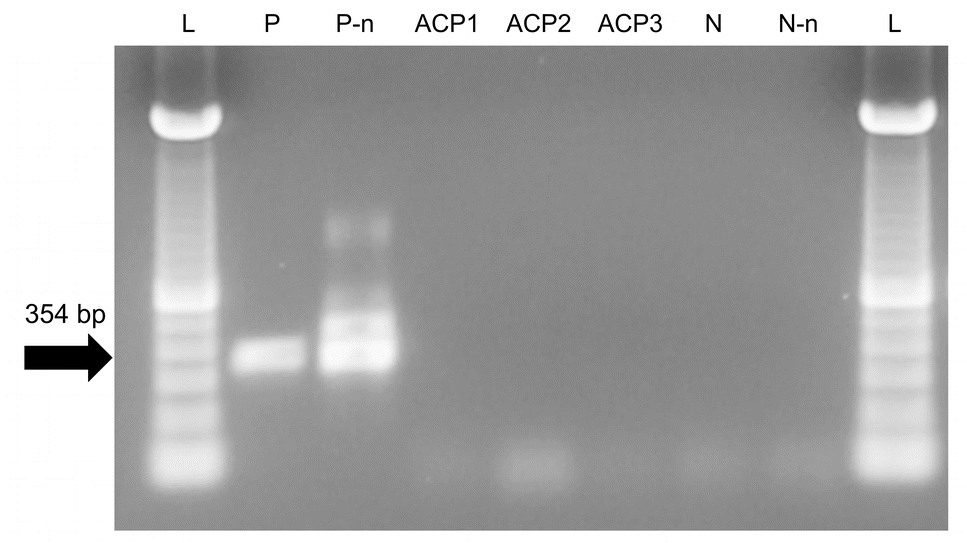

Supplement: Supplementary file 1 — Nested PCR targeting a 354 bp fragment (arrow) of the Wolbachia surface protein gene (wsp) did not detect Wolbachia in three of the field-sampled ACP adults. L: 100 bp ladder; P: positive control (genomic DNA of a Wolbachia-positive ACP) amplified using the inner primer pair only; P-n: positive control, after two rounds of amplification; ACP1-3: Wolbachia-negative ACP samples (determined by qPCR assays), after two rounds of PCR; N: no template control, tested using inner primers only; N-n: no template control, after two rounds of PCR. (GIF 104 kb) [file 248_2016_733_Fig6_ESM.gif]
