# Supplementary material for: Inter-Population Variability of Endosymbiont Densities in the Asian Citrus Psyllid (Diaphorina citri Kuwayama)
Source: Microb Ecol. 2016 Feb 4;71:999–1007. doi: 10.1007/s00248-016-0733-9 (PMC4944574; doi:10.1007/s00248-016-0733-9)
Supplement: Supplementary file 5 — (DOC 63 kb) [file 248_2016_733_MOESM3_ESM.doc]

| **Table S1 Details of endosymbiont densities in field *D. citri* with different *C*Las infection densities** | | | | | | | | | | | |  |
| --- | --- | --- | --- | --- | --- | --- | --- | --- | --- | --- | --- | --- |
| Gender | Population^a^ | *C*Las infection^b^ |  | Carsonella | |  | Profftella | |  | *Wolbachia* | | |
|  |  |  |  | Mean^c^ | Std. Error^c^ |  | Mean^c^ | Std. Error^c^ |  | Mean^c^ | Std. Error^c^ | |
| Female | LB | Negative/ND |  | 50.11 | 6.90 |  | 141.12 | 10.49 |  | 4.00 | 0.54 | |
| Female | LB | Positive |  | 43.78 | 2.80 |  | 103.05 | 8.48 |  | 3.60 | 0.65 | |
| Female | LB | Low |  | 42.92 | 3.67 |  | 114.49 | 8.24 |  | 3.64 | 0.90 | |
| Female | LB | High |  | 44.92 | 5.17 |  | 87.79 | 12.80 |  | 3.55 | 1.15 | |
| Female | LA | Negative/ND |  | 52.34 | 15.17 |  | 91.86 | 23.05 |  | 6.38 | 1.16 | |
| Female | LA | Positive |  | 43.81 | 4.43 |  | 114.39 | 18.14 |  | 4.66 | 1.01 | |
| Female | LA | Low |  | 43.03 | 7.84 |  | 85.83 | 9.47 |  | 4.81 | 1.11 | |
| Female | LA | High |  | 44.32 | 5.85 |  | 133.43 | 27.75 |  | 4.56 | 1.60 | |
| Female | CL | Negative/ND |  | 19.64 | 7.87 |  | 93.12 | 36.23 |  | 1.07 | 0.04 | |
| Female | CL | Positive |  | 21.56 | 2.21 |  | 76.01 | 9.00 |  | 1.69 | 0.33 | |
| Female | CL | Low |  | 20.75 | 2.85 |  | 81.03 | 11.70 |  | 1.48 | 0.43 | |
| Female | CL | High |  | 23.78 | 2.99 |  | 62.20 | 8.66 |  | 2.28 | 0.21 | |
| Male | LB | Negative/ND |  | 20.27 | 1.44 |  | 74.21 | 9.22 |  | 3.78 | 0.49 | |
| Male | LB | Positive |  | 16.89 | 2.10 |  | 88.51 | 15.33 |  | 4.65 | 0.32 | |
| Male | LB | Low |  | 19.43 | 2.11 |  | 105.90 | 24.00 |  | 4.89 | 0.48 | |
| Male | LB | High |  | 13.51 | 3.46 |  | 65.31 | 4.84 |  | 4.33 | 0.41 | |
| Male | LA | Negative/ND |  | 10.79 | 1.12 |  | 70.86 | 6.01 |  | 4.48 | 0.53 | |
| Male | LA | Positive |  | 12.15 | 1.46 |  | 88.15 | 9.32 |  | 4.00 | 0.74 | |
| Male | LA | Low |  | 15.38 | 2.23 |  | 126.51 | 22.32 |  | 4.48 | 2.06 | |
| Male | LA | High |  | 10.97 | 1.73 |  | 74.20 | 6.20 |  | 3.83 | 0.75 | |
| Male | CL | Negative/ND |  | 11.29 | 1.58 |  | 41.56 | 5.10 |  | 1.50 | 0.78 | |
| Male | CL | Positive |  | 15.21 | 1.32 |  | 80.77 | 8.73 |  | 1.19 | 0.24 | |
| Male | CL | Low |  | 16.20 | 1.55 |  | 79.70 | 11.18 |  | 0.87 | 0.22 | |
| Male | CL | High |  | 12.63 | 2.41 |  | 83.54 | 13.71 |  | 2.00 | 0.56 | |

^a^LB: LaBelle; LA: Lake Alfred; CL: Clermont.

^b^ND: No *C*Las detected; Low: *C*Las densities lower than 0.02 copy of *C*Las 16S rDNA per copy of *wingless*. High: *C*Las density higher than the threshold.

^c^The values were calculated from original density measurements (non-transformed values).
